# Comparative Analysis of the Lambda-Interferons IL-28A and IL-29 regarding Their Transcriptome and Their Antiviral Properties against Hepatitis C Virus

**DOI:** 10.1371/journal.pone.0015200

**Published:** 2010-12-08

**Authors:** Julia Diegelmann, Florian Beigel, Kathrin Zitzmann, Artur Kaul, Burkhard Göke, Christoph J. Auernhammer, Ralf Bartenschlager, Helmut M. Diepolder, Stephan Brand

**Affiliations:** 1 Department of Medicine II, University Hospital Munich-Grosshadern, University of Munich, Munich, Germany; 2 Department of Molecular Virology, University of Heidelberg, Heidelberg, Germany; University of Minnesota, United States of America

## Abstract

**Background:**

Specific differences in signaling and antiviral properties between the different Lambda-interferons, a novel group of interferons composed of IL-28A, IL-28B and IL-29, are currently unknown. This is the first study comparatively investigating the transcriptome and the antiviral properties of the Lambda-interferons IL-28A and IL-29.

**Methodology/Principal Findings:**

Expression studies were performed by microarray analysis, quantitative PCR (qPCR), reporter gene assays and immunoluminometric assays. Signaling was analyzed by Western blot. HCV replication was measured in Huh-7 cells expressing subgenomic HCV replicon. All hepatic cell lines investigated as well as primary hepatocytes expressed both IFN-λ receptor subunits IL-10R2 and IFN-λR1. Both, IL-28A and IL-29 activated STAT1 signaling. As revealed by microarray analysis, similar genes were induced by both cytokines in Huh-7 cells (IL-28A: 117 genes; IL-29: 111 genes), many of them playing a role in antiviral immunity. However, only IL-28A was able to significantly down-regulate gene expression (n = 272 down-regulated genes). Both cytokines significantly decreased HCV replication in Huh-7 cells. In comparison to liver biopsies of patients with non-viral liver disease, liver biopsies of patients with HCV showed significantly increased mRNA expression of IL-28A and IL-29. Moreover, IL-28A serum protein levels were elevated in HCV patients. In a murine model of viral hepatitis, IL-28 expression was significantly increased.

**Conclusions/Significance:**

IL-28A and IL-29 are up-regulated in HCV patients and are similarly effective in inducing antiviral genes and inhibiting HCV replication. In contrast to IL-29, IL-28A is a potent gene repressor. Both IFN-λs may have therapeutic potential in the treatment of chronic HCV.

## Introduction

Recently, several novel cytokines of the IL-10-like cytokine family have been discovered, including interferon (IFN)-λs [1], [2]. IFN-λs comprise three distinct genes: *IFNλ1* (*IL29*), *IFNλ2* (*IL28A*), and *IFNλ3* (*IL28B*) [1], [2]. IL-28A and IL-28B proteins are 95% identical while IL-29 shares only 80% amino acid identity with IL-28A or IL-28B. Structurally, IFN-λs are related to IL-10 and other members of the IL-10-like family such as IL-22 [3], which has recently shown to confer hepatoprotection [4]–[5]. As IL-28A, IL-28B and IL-29 functionally resemble type I IFNs (IFN-α/β), they are also considered as a novel group of IFNs (type III IFNs). Like IFN-α or IFN-β, IFN-λs exhibit activity against a broad range of viruses such as encephalomyocarditis virus (EMCV) or vesicular stomatitis virus (VSV) [1], [2], human immunodeficiency virus (HIV1) [6], Apeu virus [7], cytomegalovirus (CMV) [8], and herpes simplex virus (HSV) [9]. It has also been shown that they inhibit HCV replication [10], [11]. In contrast, we could recently demonstrate that IL-22 is not efficient against hepatitis C virus (HCV) infection [12].

IFN-λs signal through a receptor complex comprised of IL-10R2 and a unique subunit, IFNλ-R1. While IL-10R2 is widely expressed on a number of different cell types including hematopoietic cells, expression of the specific receptor IFNλ-R1 is more restricted, e.g., it seems to be weakly expressed on leukocytes. As signaling through the type-I-interferon receptor, signaling through the IFN-λ receptor results in the activation of signal transducer and activator of transcription (STAT)-1 and STAT2. Together with an accessory factor, IFN regulatory factor 9 (IRF-9; p48), STAT1 and STAT2 form the transcription factor IFN-stimulated gene factor-3 (ISGF3) which translocates to the nucleus to initiate the induction of target genes [1].

Like type I IFNs, IFN-λs are strongly induced by double stranded (ds) RNA or viral infection, suggesting common regulatory factors. In fact, it has recently been demonstrated that the *IL29* gene, similar to the gene encoding IFN-β, is regulated by virus-activated IRF3 and IRF7. In contrast, *IL28A* and *IL28B* gene expression is mainly controlled by IRF7, similar to the gene encoding IFN-α [13].

Although the antiviral effects of IL-28A and IL-29 have been compared with IFN-α, IFN-β and IFN-γ regarding their antiviral and gene-inducing activities [7], [14], [15], [16], [17], there are very limited data directly comparing signaling and antiviral properties of IL-28A and IL-29. Therefore, in this study, we directly compared these two cytokines regarding their signal transduction, target gene expression profiles, antiviral properties against HCV and their expression in different human liver diseases.

## Materials and Methods

### Reagents

Recombinant human IL-28A, IL-29 and IFN-α were purchased from R&D Systems (Minneapolis, MN). Antibodies were from BD Transduction Laboratories, Franklin Lakes, NY (pSTAT1), Upstate Biotechnology, Lake Placid, NY (pSTAT3), and Santa Cruz Biotechnology, Santa Cruz, CA (STAT1, STAT3). Horseradish peroxidase conjugated secondary antibodies to mouse or rabbit IgG and chemiluminescent substrate (SuperSignal West Dura Extended Duration Substrate) were from Pierce (Rockford, IL).

### Cell culture

The human hepatic cancer cell lines HepG2, Hep3B and Huh-7 were obtained from American Type Culture Collection (Rockville, MD) and were grown in DMEM medium with 10% fetal calf serum (PAA, Pasching, Austria), 1% penicillin/streptomycin (PAA) in a 5% CO_2_ atmosphere. Huh-7 cells containing subgenomic HCV replicons I389luc-ubi-neo/NS3-3/5.1 (Huh 5-2) were described previously [18], [19], [20], [21]. G418 (Geneticin; Gibco) was added at a final concentration of 250 µg/ml to HCV replicon-expressing cells. Primary hepatocytes from human donors were isolated and cultured as previously described [22].

### Isolation of leukocytes, peripheral blood mononuclear cells (PBMC) and granulocytes

White blood cells were isolated from fresh human anti-coagulated blood. For the isolation of total leukocytes, 5 ml of erythrocyte lysis buffer were added to 1 ml of blood. Following erythrocyte lysis and washing steps with PBS, the leukocytes were pelleted by centrifugation. For the isolation of PBMCs and granulocytes, a 6% dextran solution (molecular weight 250.000) was added to whole blood to precipitate the erythrocytes. The supernatant containing the white blood cells was treated with lysis buffer to remove any residual erythrocytes. Following washing steps, the cell suspension was layered onto a Ficoll-Hypaque density gradient and centrifuged at 400×g for 30 minutes to separate mononuclear cells from granulocytes.

### Reverse transcriptase polymerase chain reaction (RT-PCR) and quantitative PCR

Trizol reagent (Invitrogen, Karlsruhe, Germany) was used to isolate total cellular RNA. Reverse transcription of 2 µg RNA to cDNA was performed with Omniscript reverse transcriptase (Qiagen, Hilden, Germany). PCR cycling was run as follows: 40 cycles of denaturing at 95°C for 30 sec, annealing at 60°C for 30 sec, extension at 72°C for 30 sec. Real-time quantitative PCR was carried out using the Quantitect SYBR Green PCR Kit from Qiagen (Hilden, Germany) in an ABI Prism 7700 Sequence Detection System (Applied Biosystems, Darmstadt, Germany). Oligonucleotide primer pairs (MWG Biotech, Ebersberg, Germany) were designed according to the published sequences avoiding amplification of genomic DNA and are listed in Table 1.

**Table 1:** Primers used for PCR and quantitative PCR.

Gene	Primer combination
IL-10R2	5′-GGCTGAATTTGCAGATGAGCA-3′
	5′-GAAGACCGAGGCCATGAGG-3′
IFNλ-R1	5′-ACCTATTTTGTGGCCTATCAGAGCT-3′
	5′-CGGCTCCACTTCAAAAAGGTAAT-3′
IL-28	5′-AGGGCCAAAGATGCCTTAGA-3′
	5′-TCCAGAACCTTCAGCGTCAG-3′
IL-29	5′-GGACGCCTTGGAAGAGTCAC-3′
	5′-AGCTGGGAGAGGATGTGGT-3′
OAS1	5′-ATTGACAGTGCTGTTAACATCATC-3′
	5′-AGATCAATGAGCCCTGCATAAACC-3′
MX1	5′-AGATCCAGGACCAGCTGAGCCTGT-3′
	5′-GTGGAACTCGTGTCGGAGTCTGGTA-3′
GAPDH	5′-CGGAGTCAACGGATTTGGTCGTAT-3′
	5′-AGCCTTCTCCATGGTGGTGAAGAC-3′
β-actin	5′-GCCAACCGCGAGAAGATGA-3′
	5′-CATCACGATGCCAGTGGTA-3′
IL-28 (mouse)	5′-AGGGTGCCATCGAGAAGAG-3′
	5′-GTGGTCAGGGCTGAGTCATT-3′
GAPDH (mouse)	5′-CGTCCCTGAGACAAAATGGT-3′
	5′-TCTCCATGGTGGTGAAGACA-3′

### Luciferase assay

HepG2 cells were transiently transfected with either a −970 nt human OAS1 promoter - luciferase construct or a −553/+10 human MX1 promoter - luciferase construct, both in a pGL2-BV vector, using Superfect® (Qiagen, Hilden, Germany), as recently described [23]. Transfection efficiency was verified by β-galactosidase assay in all experiments.

### Gel electrophoresis and immunoblotting

Cells were solubilized in lysis buffer consisting of 20 mM Tris-HCl (pH 7.4), 150 mM NaCl, 2 mM EDTA, 2 mM EGTA, 1% Nonidet P-40, 2 mM phenylmethylsulfonyl fluoride, a protease inhibitor cocktail (Roche, Mannheim, Germany) and phosphatase inhibitors (400 mM sodium orthovanadate and 4 mM NaF). Cell lysates were passed six times through a 21 G needle. After chilling on ice for 30 minutes, lysates were cleared by centrifugation for 20 minutes at 10,000 g. The Bradford method was used to quantify the protein concentration of each sample. Immunoblotting was performed as previously described [24].

### Anti-HCV assay in Huh 5-2 cells

Huh 5-2 cells (“HCV-Huh-7”) were seeded in 6-well plates at a density of 2×10^5^ per well in complete DMEM. Following incubation for 24 hours at 37°C (5% CO_2_), medium was replaced with 2 ml DMEM supplemented with IL-28A, IL-29 or IFN-α. After further incubation at 37°C for 72 hours, cell culture medium was removed and luciferase activity was determined using a Lumat LB9507 luminometer (Berthold, Freiburg, Germany) as described recently [21].

### IL-28A immunoluminometric assay (ILMA)

For quantification of IL-28A in human serum samples, Human IL-28A/IFN-lambda 2 DuoSet (R&D Systems, Wiesbaden, Germany) was used to develop an IL-28A-specific immunoluminometric assay. The detection limit was 4.9 pg/ml. Signal detection was performed with a biotinylated detection antibody and incubation with neutravidin-HRP and the chemoluminescent substrate Femtoglow (Michigan Diagnostics, Troy, MI).

### Murine cytomegalovirus (MCMV) infection in vivo

1×10^6^ plaque-forming units of MCMV of the Smith strain [25] in PBS were injected intravenously into C57/BL6 mice as described previously [8]. Control mice got an injection of PBS only. After 45 h, mice were euthanized by CO_2_ inhalation, and the livers were collected and homogenized in Trizol reagent to isolate total RNA. The study was approved by the Animal Care and Use Committee of the State of Bavaria (Regierung von Oberbayern, approval ID 209.1/211-2531-18/03) according to the National Institutes of Health “Guide for the Care and Use of Laboratory Animals”.

### Sampling of human liver biopsy tissue, blood and serum samples including Ethics statement

Human liver biopsy tissue was obtained from patients undergoing diagnostic liver biopsy for medical reasons such as staging of chronic hepatitis C. The study was approved by the Ethics committee of the Ludwig-Maximilians-University Munich (Department of Medicine, Munich-Grosshadern) and adhered to the ethical principles for medical research involving human subjects of the Helsinki Declaration (http://www.wma.net/e/policy/b3.htm). All participating patients gave written, informed consent prior to liver biopsy sampling. A 3 mm long piece of the biopsy cylinder was immediately stored in Trizol reagent and RNA was isolated as described previously [26]. Human blood and serum samples were obtained after written informed consent from patients and controls and were stored at −80°C until further analysis.

### Microarray analysis

After reaching 70% confluency, Huh-7 cells were incubated overnight with serum-reduced medium containing 1% FCS. The next day, cells were stimulated in triplicates with 100 ng/ml IL-28A, IL-29 or left unstimulated. RNA was isolated at the indicated time points using the RNeasy kit from Qiagen (Hilden, Germany). For the analysis of the cytokine-induced gene expression, Agilent Whole Human Genome Oligo Microarrays were used in combination with a One-Color based hybridization protocol. Signals on the microarrays were detected with the Agilent DNA Microarray Scanner. Differential gene expression was identified within the human cells by applying appropriate biostatistics to the data set. GeneSpring GX 10 analysis software (Agilent Technologies, Santa Clara, CA) was used to normalize and analyze the raw data. Cytokine-induced gene expression was calculated in comparison to unstimulated cells at the same time points. Welch's approximate t-test (“unpaired unequal variance”, parametric) was applied to the comparison of the different groups. Resulting p-values were corrected for multiple testing using the algorithm of Benjamini and Hochberg [27]. Functional analysis (categories of biological processes, molecular functions and pathway categories) of induced and repressed genes was performed using the Panther software [28]. By comparing cytokine-regulated gene identification numbers (IDs) to the distribution of all gene IDs represented on the Whole Human Genome Oligo Microarray (Agilent Technologies), it was calculated whether a specific class is over- or underrepresented. P-values of p<10^−5^ (based on binomial test) were considered as a sign of manifest enrichment in the context of a Panther analysis for biological processes, molecular functions and pathway categories. All microarray data presented are MIAME compliant and the raw data have been deposited in a MIAME compliant database in MIAMExpress (available at www.ebi.ac.uk/microarray/, accession number E-MEXP-2861) as detailed on the MGED Society website http://www.mged.org/Workgroups/MIAME/miame.html.

### Statistical analysis

Statistical analysis was performed by using two-tailed Student's t-test. P levels<0.05 were considered as statistically significant. Standard errors of the mean (SEM) were calculated by dividing the standard deviation (SD) by the square root of the number of single data in the respective group.

## Results

### Hepatic cells express the IFN-λ receptor complex

**Figure 1:**
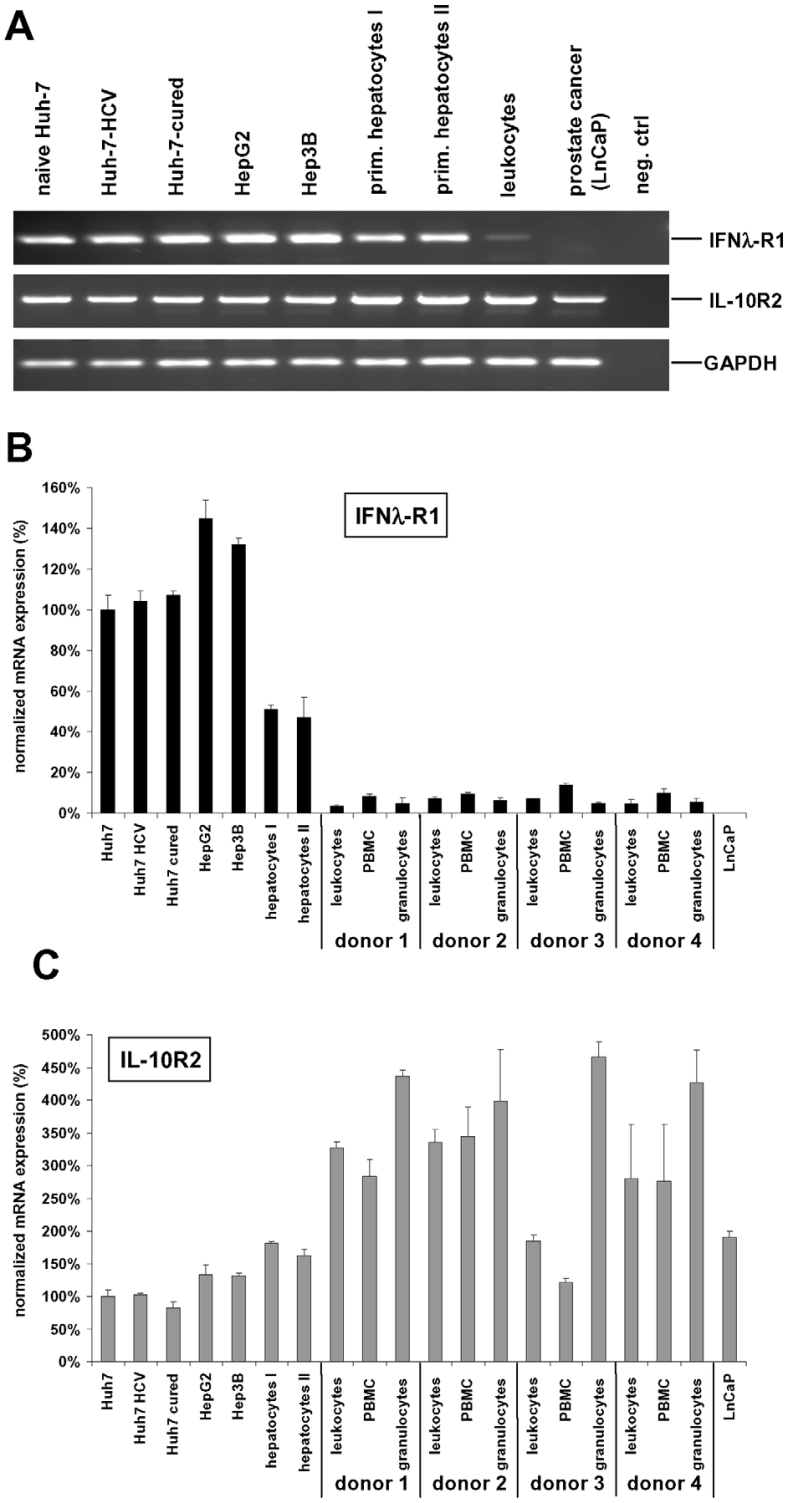
The IFN-λ receptor complex consisting of IL-10R2 and IFNλ-R1 is expressed in hepatic cell lines. (A) Expression of IL-10R2 and IFNλ-R1 in various hepatic cell lines, primary hepatocytes from two different donors and primary leukocytes were analyzed by RT-PCR analysis of mRNA derived from cells as indicated. The prostate cancer cell line LnCaP was used as a negative control for IFNλ-R1 expression. (B) Quantitative PCR analysis reveals a much lower expression of IFNλ-R1 mRNA in leukocytes from four different donors in comparison to liver cells. PBMC express twice as much IFN-λR1 than granulocytes. Expression data are normalized to Huh-7 cells. (C) IL-10R2 was expressed at higher levels in leukocytes in comparison to hepatic cells. Data are normalized to the expression in Huh-7 cells.

In order to utilize a hepatic cell model to study the IFN-λ ligand-receptor system, we first confirmed that the IFN-λ receptor subunits IL-10R2 and IFNλ-R1 are present in hepatic cells. RT-PCR analysis demonstrated IL-10R2 and IFNλ-R1 mRNA expression in several human hepatic cancer derived cell lines (HepG2, Hep3B, Huh-7) as well as in HCV replicon expressing Huh-7 5-2 cells and Huh-7 cells cured from HCV by IFN-α and IFN-γ (HCV-cured Huh-7) (Fig. 1A). Primary hepatocytes from two different donors also expressed mRNA for both IFN-λ receptor subunits (Fig. 1A) while leukocytes show only low expression (Fig.1A). The prostate cancer cell line LNCaP was used as a negative control for IFNλ-R1 expression. Quantitative PCR analyses including total leukocytes as well as peripheral blood mononuclear cells (PBMC) and granulocytes from four different donors revealed that leukocytes express only 5.5±1.8% of the level or IFN-λR1 in comparison to Huh-7 cells (Fig. 1B). Among the leukocytes, expression of IFN-λR1 was 1.9±0.5-fold higher in PBMC than in granulocytes (Fig. 1B). IL-10R2 mRNA expression was higher in leukocytes compared to liver cells (Fig. 1C), and granulocytes had a 1.7±0.1-fold higher IL-10R2 expression than PBMC.

### IFN-λs induce STAT1 but not STAT3 phosphorylation

**Figure 2:**
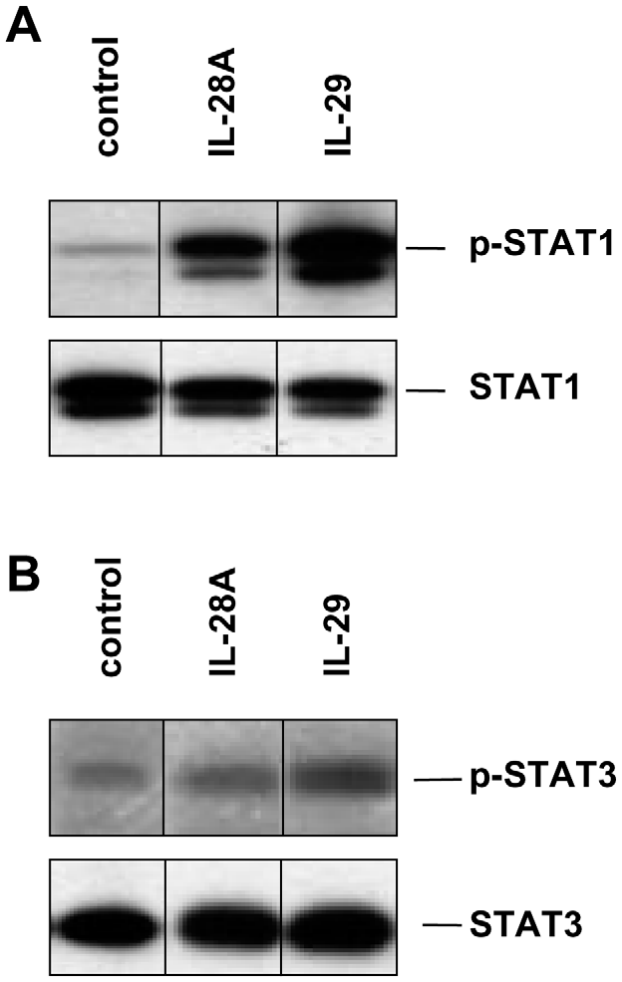
IL-28A and IL-29 strongly activate STAT1 but not STAT3 in naïve Huh-7 cells. Activation and expression of phospho-STATs and protein loading of the respective STAT proteins were assessed by immunoblotting. (A) Phospho-STAT1 activation after 15 min of stimulation with IL-28A and IL-29 (100 ng/ml). (B) STAT3 is phosphorylated only to a low extent by stimulation with IL-29 but not with IL-28A. One representative experiment (n = 3) is shown.

Previous studies in other cell systems reported activation of STAT signaling by IFN-λs [1], [14]. Therefore, we investigated the influence of IL-28A and IL-29 on phosphorylation levels of STAT1 and STAT3 in naïve Huh-7 cells. As demonstrated in Fig. 2A, both cytokines activated STAT1 whereas IL-29 had a stronger effect than IL-28A. On the other hand, STAT3 was not activated by IL-28A and stimulation with IL-29 had only minor effects (Fig. 2B).

### IL-28A and IL-29 induce expression of similar genes in Huh-7 cells but differ in their gene down-regulating abilities

Next, we analyzed by microarray experiments the IFN-λ-induced gene expression in hepatic cells. Naïve Huh-7 cells were stimulated for 6 hours with 100 ng/ml IL-28A or IL-29, while controls were left unstimulated for the same time interval. Altogether, a total number of 389 genes were influenced equal to or more than two-fold by IL-28A (117 genes up-regulated, 272 down-regulated) and a total number of 115 genes by IL-29 (111 genes up-regulated, 4 down-regulated), respectively (p<0.01 without correction for multiple testing) (Table 2). When applying more stringent criteria (adjusted p-values [adj.-p]<0.05; corrected for multiple testing by the Benjamini and Hochberg algorithm [27]), a total of 154 genes was significantly regulated by IL-28A (65 genes up-regulated, 89 down-regulated) and only 3 genes were significantly regulated by IL-29 (all up-regulated). The top 20 hits of up-regulated genes were identical for both cytokines although in a slightly different order (Table 3). The gene that was most induced by both cytokines was *MX1* which was increased 167.0- and 183.4-fold by IL-28A and IL-29, respectively.

**Table 2:** Summary for the number of up- and down-regulated genes after 6 hours applying different stringency criteria.

	p≤0.01			adj.-p≤0.05		
	FC≥2			FC≥2		
	up	down	Σ	up	down	Σ
**IL-28A vs. untreated**	117	272	389	65	89	154
**IL-29 vs. untreated**	111	4	115	3	0	3

The left group represents hits from the initial screening with uncorrected p-value of ≤0.01 vs. untreated cells. The right group depicts the number of regulated genes after the p-value was adjusted for multiple testing. FC = fold-change.

**Table 3:** Overview of the 20 genes whose expression was most strongly induced (p<0.01) by IL-28A and IL-29 in Huh-7 cells after 6 hours of stimulation.

Gene ID	Gene symbol	Description	IL-28A treatment fold increase	IL-29 treatment fold increase	Ratio IL-28A/IL-29
NM_002462	MX1	Homo sapiens myxovirus (influenza virus) resistance 1	167.0	183.4	0.91
NM_001548	IFIT1	Homo sapiens interferon-induced protein with tetratricopeptide repeats	89.7	99.8	0.90
NM_022168	IFIH1	Homo sapiens interferon induced with helicase C domain 1	48.3	58.7	0.82
NM_002534	OAS1	Homo sapiens 2′,5′-oligoadenylate synthetase 1	32.2	41.0	0.79
NM_022873	IFI6	Homo sapiens interferon, alpha-inducible protein 6 (6–16)	28.5	31.8	0.90
NM_003733	OASL	Homo sapiens 2′,5′-oligoadenylate synthetase-like	27.2	35.5	0.77
NM_004335	BST2	Homo sapiens bone marrow stromal cell antigen 2 (Tetherin)	26.0	28.2	0.92
NM_001549	IFIT3	Homo sapiens interferon-induced protein with tetratricopeptide repeats 3	25.1	29.4	0.85
BG547557	CMPK2	Homo sapiens cytidine monophosphate (UMP-CMP) kinase 2, mitochondrial	21.9	22.1	0.99
NM_003641	IFITM1	Homo sapiens interferon induced transmembrane protein 1 (9–27)	20.9	26.8	0.78
NM_033255	EPSTI1	Homo sapiens epithelial stromal interaction 1 (breast)	18.5	19.4	0.95
NM_017631	DDX60	Homo sapiens DEAD (Asp-Glu-Ala-Asp) box polypeptide 60	18.1	20.4	0.89
NM_006187	OAS3	Homo sapiens 2′-5′-oligoadenylate synthetase 3	17.7	19.7	0.90
NM_006084	IRF9	Homo sapiens interferon-stimulated transcription factor 3, gamma	17.1	15.2	1.13
NM_001547	IFIT2	Homo sapiens interferon-induced protein with tetratricopeptide repeats 2	15.3	19.3	0.79
NM_017654	SAMD9	Homo sapiens sterile alpha motif domain containing 9	15.0	16.7	0.90
NM_000593	TAP1	Homo sapiens transporter 1, ATP-binding cassette, sub-family B (MDR/TAP)	12.1	14.6	0.83
NM_030641	APOL6	Homo sapiens apolipoprotein L 6	11.5	12.3	0.93
NM_080657	RSAD2	Homo sapiens radical S-adenosyl methionine domain containing 2	10.3	13.9	0.74
NM_005101	ISG15	Homo sapiens ISG15 ubiquitin-like modifier	9.4	10.8	0.87

Furthermore, IL-28A stimulation resulted in an at least two-fold decreased expression of a multitude of genes (272 genes for p<0.01 without multiple testing adjustment; 89 genes for adj.-p<0.05) with up to 9.3-fold reduced levels (Table 4).

**Table 4:** Overview of the 20 genes whose expression was most strongly repressed (p<0.01) by IL-28A in Huh-7 cells after 6 hours of stimulation.

Gene ID	Gene symbol	Description	IL-28A treatment fold decrease
NM_018027	FRMD4A	Homo sapiens FERM domain containing 4A	−9.33
NM_000721	CACNA1E	Homo sapiens calcium channel, voltage-dependent, R type, alpha 1E subunit	−8.82
X94553	FOXE1	Homo sapiens forkhead box E1 (thyroid transcription factor 2)	−8.37
THC2559929	SNC73	Q9UP60_HUMAN (Q9UP60) SNC73 protein	−7.65
NM_080819	GPR78	Homo sapiens G protein-coupled receptor 78	−5.47
NM_198390	CMIP	Homo sapiens c-Maf-inducing protein	−5.36
AB014771	MOP-1	Homo sapiens MOP-1	−5.16
THC2678411	THC2678411	Q34Z38_9GAMM (Q34Z38) Outer membrane efflux protein precursor	−4.84
AF490258	FBRSL1	Homo sapiens fibrosin-like 1	−4.74
NM_021569	GRIN1	Homo sapiens glutamate receptor, ionotropic, N-methyl D-aspartate 1	−4.51
NM_032805	ZSCAN10	Homo sapiens zinc finger and SCAN domain containing 10	−4.32
Y10152	Y10152	Homo sapiens CRF2 receptor, beta isoform, aberrantly spliced, (94bp deletion)	−4.44
NM_004054	C3AR1	Homo sapiens complement component 3a receptor 1	−4.26
NM_003961	RHBDL1	Homo sapiens rhomboid, veinlet-like 1 (Drosophila)	−4.22
NM_019105	TNXB	Homo sapiens tenascin XB (TNXB), transcript variant XB	−4.08
NM_013271	PCSK1N	Homo sapiens proprotein convertase subtilisin/kexin type 1 inhibitor	−3.98
NM_033120	NKD2	Homo sapiens naked cuticle homolog 2 (Drosophila)	−3.90
NM_001003845	SP5	Homo sapiens Sp5 transcription factor	−3.72
NM_000554	CRX	Homo sapiens cone-rod homeobox	−3.71
AF335478	KLK3	Homo sapiens prostate-specific antigen variant 2	−3.60

We then analyzed the induced and repressed genes for specific enrichment of defined biological processes and molecular functions using the Panther database [28]. The genes activated by IL-28A and IL-29 comprised genes of immunity and defense (p = 5.7×10^−17^ for IL-28A, p = 2.5×10^−16^ for IL-29 vs. distribution of all genes on the microarray chip), especially interferon-mediated antiviral immunity (p = 2.1×10^−7^ for IL-28A, p = 1.8×10^−6^ for IL-29) and proteolysis (IL-28A only, p = 3.8×10^−6^) (Table 5; Fig. S1A). Genes of the molecular function class of ligase (p = 3.7×10^−6^ for IL-28A, p = 8.2×10^−7^ for IL-29) and its subgroup ubiquitin protein ligase (p = 8.6×10^−7^ for IL-28A, p = 1.1×10^−6^ for IL-29) were significantly enriched (Table 5; Fig. S1B).

**Table 5:** Functional classification of IL-28A and IL-29-induced and repressed genes regarding the categories of biological processes and molecular functions applying the Panther software [28].

**Enrichment class:** **biological processes**	**induced by IL-28A** **(p-value)**	**induced by IL-29** **(p-value)**
Immunity and defense	1.8×10^−6^	2.1×10^−7^
Interferon-mediated immunity	5.7×10^−17^	2.5×10^−6^
Proteolysis	3.8×10^−6^	n. s.
**Enrichment class:** **biological processes**	**downregulated by IL-28A** **(p-value)**	**downregulated by IL-29** **(p-value)**
mRNA transcription regulation	1.2×10^−6^	n. s.
**Enrichment class:** **molecular function**	**induced by IL-28A** **(p-value)**	**induced by IL-29** **(p-value)**
Ligase	3.7×10^−6^	8.2×10^−7^
Ubiquitin protein ligase	8.6×10^−7^	1.1×10^−6^
**Enrichment class:** **molecular function**	**downregulated by IL-28A** **(p-value)**	**induced by IL-29** **(p-value)**
Nucleic acid binding	1.3×10^−7^	n. s.
Other DNA-binding protein	2.5×10^−9^	n. s.
Homeobox transcription factor	2.3×10^−7^	n. s.

In all classifications, p-values <10^−5^ vs. the distribution of all genes on the microarray chip were considered as significant enrichment. n.s.: not significant.

Amongst the downregulated genes following IL-28A stimulation, those involved in the biological process of mRNA transcription regulation were especially enriched (p = 1.2×10^−6^; Table 5; Fig. S2A). Additionally, analysis revealed a down-regulation in the molecular function class of nucleic acid binding proteins (p = 1.3×10^−7^) with its subclass “other DNA-binding proteins” (p = 2.5×10^−9^; Table 5; Fig. S2B). Moreover, homeobox transcription factors, a subclass of the transcription factor group, were significantly enriched (p = 2.3×10^−7^; Table 5 and Fig. S2B).

In contrast, IL-29 reduced gene expression of only four genes more than two-fold (p<0.01; data not shown). However, when corrected for multiple testing, none of these regulations remained significant (adj.-p>0.05; data not shown).

In addition, we analyzed gene expression after three hours of stimulation with IL-28A or IL-29, respectively. The overall number of genes regulated more than two-fold was much lower than after 6 hours (IL-28A: 16 genes up-regulated, 0 down-regulated; IL-29: 39 genes up-regulated, 14 down-regulated). After correction for multiple testing, only two genes remained significantly up-regulated by IL-29 (*MX1* and *IFIT1*) while IL-28A had no significant effect on gene expression (data not shown).

### Validation of the microarray data by quantitative PCR and luciferase assay

In the next set of experiments, we verified the IFN-λ induced gene expression in naïve Huh-7 cells by quantitative RT-PCR in an independent set of RNA samples at different time points. In this analysis, we included the most strongly induced genes for both cytokines (*MX1*; Table 3) and additionally analyzed expression of *OAS1*, coding for another important antiviral protein (2′,5′-OAS) regulated by IL-28A and IL-29 (32.2- and 41.0-fold, respectively; Table 3).

**Figure 3:**
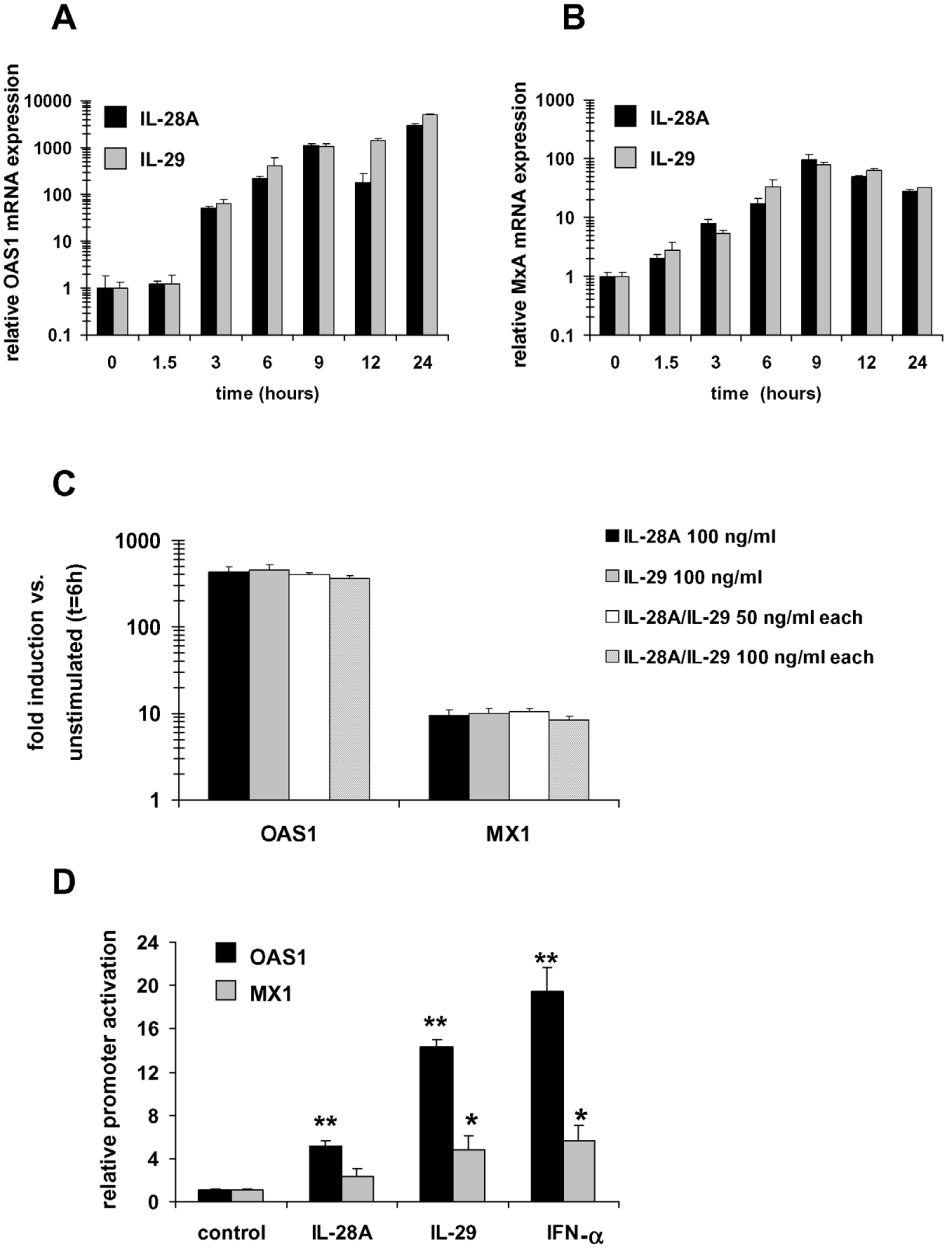
IL-28A and IL-29 induce OAS1 and MX1 mRNA expression and promoter activity in hepatic cells. (A) OAS1 mRNA expression is increased by IL-28A and IL-29 stimulation of Huh-7 cells reaching maximal levels after 24 hours. Samples were analyzed by quantitative PCR in triplicate for each group and were normalized to the expression in unstimulated cells. (B) IL-28A and IL-29 induce maximal MX1 mRNA expression after 9 hours of stimulation as determined by qPCR and normalized to untreated cells. (C) IL-28A and IL-29 do not act synergistically. OAS1 and MX1 gene induction by IL-28A and IL-29 alone (100 ng/ml) was not significantly different from treatment with both cytokines together (50 or 100 ng/ml each) in Huh-7 cells. (D) Reporter gene assays and OAS1 luciferase construct revealed activation of the OAS1 promoter 5.1-fold (IL-28A) and 14.3-fold (IL-29), respectively, and the MX1 promoter 2.4-fold (IL-28A) and 4.8-fold (IL-29), respectively, in HepG2 cells following stimulation for 6 hours with 100 ng/ml IL-28A or IL-29, respectively. IFN-α was used as a positive control. Baseline reporter gene activity was set as 1. IL-28A/IL-29 induced reporter gene activity in all other groups was calculated as –fold increase in comparison to this control group. *p<0.05; **p<0.0005 vs. control.

The results are depicted in Figure 3 and show a significant increase in OAS1 mRNA expression of more than 2800-fold following IL-28A and more than 5000-fold by IL-29 stimulation after 24 hours (Fig. 3A). MX1 mRNA expression was induced up to 95-fold by IL-28A and 78-fold by IL-29 (Fig. 3B). While MX1 reached maximal expression levels after 9 hours, OAS1 was induced most strongly after 24 hours (Fig. 3A, B).

To analyze possible additive/synergistic effects of IL-28A and IL-29, Huh-7 cells were stimulated with either cytokine alone (at a concentration of 100 ng/ml) or with a combination of both cytokines together (50 ng/ml each and 100 ng/ml each). As shown in Figure 3C, no significant difference was observed between the different treatments.

We next aimed to confirm our data in another hepatic cell line (HepG2) using a luciferase promoter assay as an additional experimental approach to determine the influence of IFN-λs on the transcriptional regulation of these two antiviral genes. Promoter activity of a human –970 nt *OAS1* promoter-luciferase construct and of a −553/+10 human MX1 promoter-luciferase construct were examined, following incubation of HepG2 cells with 100 ng/ml IL-28A and IL-29. OAS1 promoter activity was significantly stimulated 5.1-fold and 14.3-fold, respectively, above baseline by treatment with IL-28A and IL-29 for 6 hrs (Fig. 3D; p<0.0005). MX1 promoter activity was increased 2.4-fold and 4.8-fold by IL-28A and IL-29, respectively (Fig. 3D; p<0.05).

### IFN-λs decrease HCV replication in vitro

**Figure 4:**
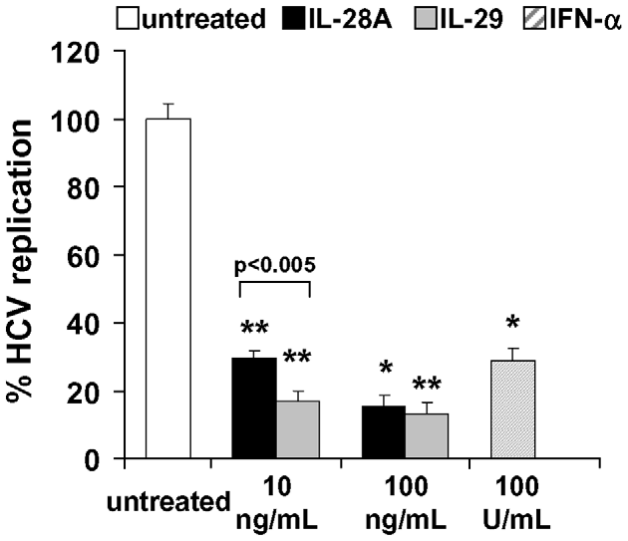
IL-28A and IL-29 significantly decrease HCV replication. In luciferase assays with HCV replicon expressing cells, 10 or 100 ng/mL IL-28A or IL-29 strongly reduced HCV replication up to 84.8% and 87.7%, respectively, compared to untreated cells (p<5×10^−14^; IFN-α: 72.5% reduction vs. untreated, p<5×10^−13^).

To investigate whether the activation of genes encoding the antiviral proteins 2′,5′-OAS and MX1 results in antiviral activity *in vitro*, we analyzed the effect of IL-28A and IL-29 on the HCV replication rate in HCV replicon expressing Huh-7 cells. In these experiments, both IL-28A and IL-29 (100 ng/ml) significantly decreased HCV replication in Huh-7 cells by 84.8% and 87.7%, respectively (**p<9×10^−15^ vs. untreated; IFN-α: 72.5% reduction vs. untreated, **p<5×10^−13^, Fig. 4). At a cytokine concentration of 10 ng/ml, IL-29 had significantly stronger inhibitory effects on HCV replication (reduction of 83.4%) than IL-28A (reduction of 70.1%; p<0.005).

### IL-28A and IL-29 mRNA and protein expression is increased in the liver tissue and serum of patients with HCV infection

**Figure 5:**
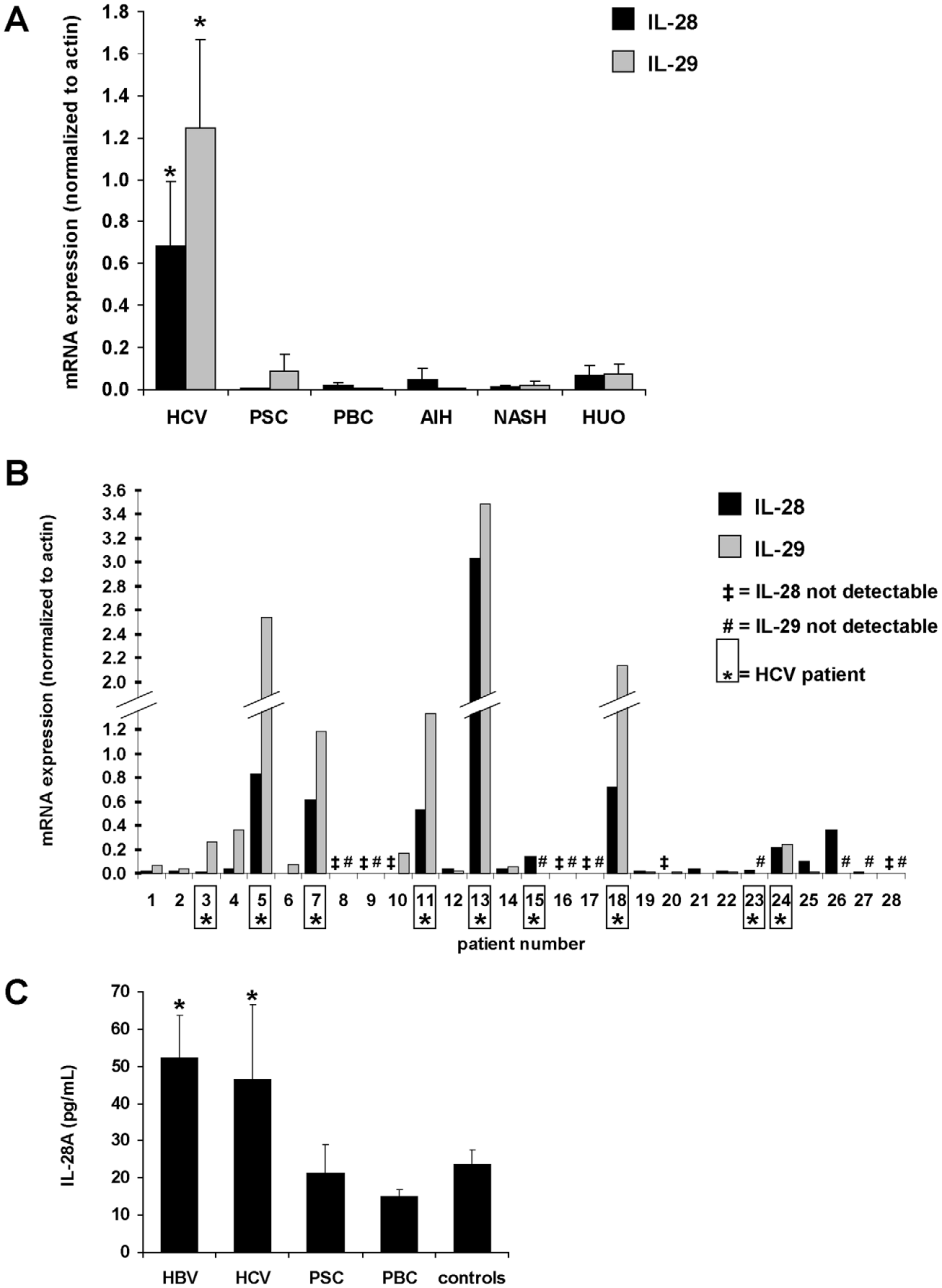
Expression of IL-28A and IL-29 is significantly increased in human hepatitis C infection. (A) Total IL-28A/B expression in liver biopsies from HCV patients (n = 9) is 18.4-fold higher in comparison to patients with other liver diseases such as primary sclerosing cholangitis, (PSC; n = 2), primary biliary cholangitis (PBC; n = 2), autoimmune hepatitis (AIH; n = 2), non-alcoholic steatosis hepatis (NASH; n = 3), or hepatitis of unknown origin (HUO; n = 9) as determined by quantitative PCR. IL-29 expression was 26.4-fold higher in HCV vs. non-HCV biopsies (*p<0.05 vs. any other group). (B) Detailed analysis of each single biopsy reveals expression of total IL-28 in all 9 out of 9 HCV patients ( = number marked by an asterisk) while in 8 out of 19 non-HCV biopsies, IL-28 could not be detected after 40 PCR cycles (‡ = IL-28 not detectable; p<0.05). IL-29 was expressed in 7 out of 9 HCV biopsies and in 12 out of 19 non-HCV biopsies (# = IL-29 not detectable). (C) Analysis of IL-28A serum levels in liver disease patients and controls by an IL-28A-specific ILMA demonstrated significantly higher IL-28A protein expression in the sera of HBV and HCV patients in comparison to controls or PBC patients (*p<0.05; n = 15 in each group [except PBC: n = 24]).

Having shown that IFN-λs inhibit HCV replication, we next analyzed IFN-λ expression in viral infection *in vivo*. First, we measured total IL-28 and IL-29 mRNA levels in liver biopsies (n = 28) from patients with HCV and other non-viral liver diseases. IL-28 mRNA expression was highest in HCV patients (18.4-fold vs. other liver diseases, p<0.05; Fig. 5A) and was detectable in 100% (9/9) of HCV biopsies but in only 58% (11/19) of biopsies from non-HCV hepatitis (p<0.05; Fig. 5B). Similarly, IL-29 mRNA expression was highest in HCV patients (26.4-fold vs. non-HCV liver diseases, p<0.05; Fig. 5A) and was detectable in 78% (7/9) of HCV biopsies but in only 63% (12/19) of biopsies from non-HCV hepatitis (Fig. 5B). In all biopsies, IL-28 and IL-29 mRNA expression correlated highly with each other (r = 0.892).

We then measured IL-28A serum protein concentration using an immunoluminometric assay (ILMA) in another group of liver disease patients, each comprising 15 patients with HCV or HBV infection, primary sclerosing cholangitis (PSC), and 24 patients with primary biliary cirrhosis (PBC) as well as 15 controls. IL-28A serum protein levels were significantly higher in patients with viral infection (mean concentrations of 52.2 pg/ml in HCV and 46.3 pg/ml in HBV patients) in comparison to non-viral liver diseases such as PBC (mean concentration 14.9 pg/ml; p<0.01 vs. HCV/HBV), PSC (mean concentration 21.2 pg/ml; p<0.05 vs. HCV/HBV) or a control group (mean concentration 23.6 pg/ml; p<0.05 vs. HCV/HBV) (Fig. 5C).

### IL-28 mRNA expression is increased in the liver of murine cytomegalovirus (MCMV)-infected mice

**Figure 6:**
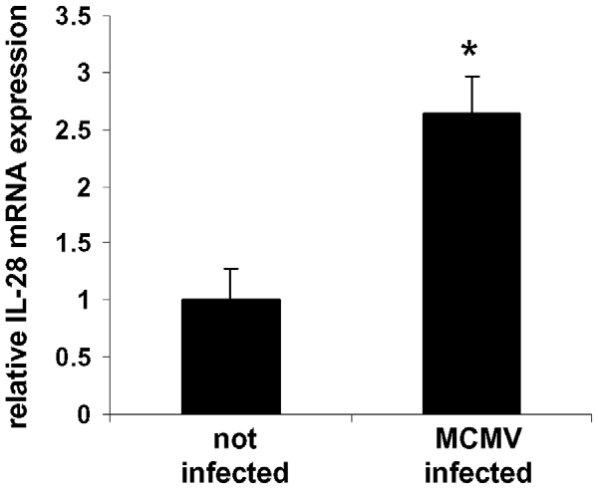
IL-28 expression is increased in murine viral hepatic infection. Mice were infected with 1×10^6^ pfu of murine cytomegalovirus for 45 hours. Quantitative PCR analysis demonstrated an increase of IL-28 expression of 2.7-fold in comparison to not infected control mice. * p<0.005 vs. not infected. No IL-29 gene has been described in mice, therefore not allowing expression analysis of this cytokine.

Given the current lack of a simple murine model for HCV infection [29] and in order to analyze if the up-regulation of IFN-λs *in vivo* can be found in viral liver disease other than HCV and HBV, we studied IFN-λ expression in murine cytomegalovirus (MCMV) infection, an established model of murine viral hepatitis [30], [31]. Given that no IL-29 gene is known in mice, we solely determined IL-28 mRNA expression levels 45 hours after infection. Compared to non-infected mice (n = 4), IL-28 mRNA expression was 2.7-fold higher in MCMV-infected mice (n = 10; p<0.005; Fig. 6).

## Discussion

This study represents the first detailed comparative investigation of IL-28A- and IL-29-mediated biological activities and gene expression patterns in viral hepatitis and non-viral liver disease *in vivo*. We demonstrate that the IFN-λ receptor complex is functionally expressed in liver cells while IFN-λR1 is expressed only at low levels in leukocytes. Both IL-28A and IL-29 are able to induce STAT1 phosphorylation in hepatic cells with IL-29 showing slightly stronger effects. Our microarray analysis revealed activation of mostly identical genes by IL-28A and IL-29. Among them were numerous genes involved in interferon-mediated immunity and antiviral defense, such as *MX1* [32], *OAS1*, *OAS3* and *OASL* [33], *BST2* (tetherin) [34], inhibitors of protein synthesis such as *PKR* [35], *IFIT1* and *IFIT2* [36], antiproliferative genes like *IFITM1* [37] and the Bcl-2-related proapoptotic gene *APOL6* [38], *TAP1* (involved in antigen processing and presentation) [39], as well as other interferon-stimulated genes such as *ISG15* (ubiquitin-like modifier) [33] or *IFI6* (6–16 protein) [40]. A recent study analyzing the effect of IL-29 in HepG2 cells revealed 35 up-regulated genes following IL-29 stimulation [41]. Among these, 27 were found in our analysis of IL-28A as well as IL-29 confirming the similarity between these two cytokines.

The analogy of IL-28A and IL-29 is further supported by the fact that we observed no synergistic or additive effect of both cytokines concerning the induction of gene expression. This suggests common signaling pathways and functions of these cytokines. Moreover, the dose of IFN-λ used in this study (100 ng/ml) seems to be a saturation concentration for this cytokine.

IFN-λs also increased mRNA expression of STAT1, STAT2 and IRF9 whose protein products together form the ISGF3 transcription factor complex characteristic for IFN type I and type III signaling. ISGF3 in turn activates a number of IFN-stimulated genes (ISG), thereby contributing to the antiviral response. Similarly, a recent study showed that IL-29 stimulation also leads to increased levels of total STAT1 and STAT2 protein and hence to a prolonged induction of target genes [14].

Three additional genes with relevant immunological functions which were up-regulated by IFN-λs included *TLR3, MDA5 (IFIH1)* and *RIG-I (DDX58)*. The proteins belong to the pattern recognition receptors (PRRs) of the innate immune system and bind specifically extracellular derived dsRNA (TLR3) and cytoplasmic viral RNA (MDA5, RIG-I) [42]. It has recently been demonstrated that TLR3 ligands mediate an antiviral state against HCV in hepatic cells [43] and induce antiviral activity of IFN-λs [44]. RIG-I is likewise important for the antiviral state in HCV infection [45]. On the other hand, HCV is able to inhibit several PRR pathways [46], suggesting that up-regulation of PRR mRNA expression by IL-28A and IL-29 might counteract this HCV-mediated effect. In our study, IL-28A and IL-29 up-regulated a nearly identical gene transcription program which also resembles that of IFN-α [41]. However, it has recently been demonstrated that neither IFN-α nor IL-29 are able to down-regulate gene expression in hepatic cells [16], [17], [41]. In concordance with these previous studies [16], [17], [41], we measured no significant down-regulation of genes following IL-29 stimulation.

Therefore, it is of great interest that in contrast IL-28A significantly reduced the expression of 89 genes more than 2-fold (adj.-p<0.05) in our experiments. This number was even higher than the number of induced genes (65, adj.-p<0.05). Many of these genes code for DNA-binding proteins and are involved in the transcriptional regulation.

The activation of antiviral proteins by IL-28A and IL-29 tempted us to investigate the effect of IFN-λs on the replication rate of HCV in an *in vitro* system expressing HCV replicons. In these experiments, we demonstrated that both, IL-28A and IL-29 at a dose of 100 ng/ml reduce significantly the replication rate of HCV with the same efficacy and comparable to IFN-α. However, at a concentration of 10 ng/ml, IL-29 is 20% more effective in inhibiting HCV replication than IL-28A (p<0.005). Currently, a pegylated form of IL-29 is tested in a phase 1b clinical study in HCV patients [47]. Preliminary results indicate that it is effective in reducing viral load without typical side effects seen with IFN-α [47] which may be related to the more restricted expression of the IFN-λ receptor subunit IFN-λR1 compared to the IFN-α receptor subunits IFNAR1 and IFNAR2.

A recent study demonstrated that IL-28B appears to be the most potent IFN-λ cytokine, at least in EMCV infection [48]. However, in VSV infection, IL-28B did not show any effect [48]. Moreover, there were also considerable differences in specific activities between the same cytokines derived from different sources [48] indicating that the production and preparation methods are crucial variables. Interestingly, several recent publications describe an association between single nucleotide polymorphisms (SNPs) in the *IL28B* gene region and the clearance of HCV infection, either naturally occurring [49] or induced by treatment with a combination therapy of IFN-α and ribavirin [50], [51], [52]. Some of these SNPs are located in the *IL28B* gene itself while others are situated upstream or downstream of *IL28B* [16], [17], [41], [49]. The functional consequences of the SNPs in the *IL28B* gene region are not yet clear and need further investigations. Given that the *IL28A* and *IL28B* genes lie in close proximity on chromosome 19q13.13, it is possible that some of these SNPs influence regulatory elements of both *IL28A* and *IL28B* [51]. This is supported by data demonstrating lower *IL-28A/B* mRNA expression in whole blood and PMBCs, respectively, in minor allele carriers and non-responders to IFN-α therapy [51], [52]. As *IL28B* mRNA is 98% identical to *IL28A* mRNA and can hardly be distinguished from the latter by PCR analysis, it cannot be excluded that IL-28A also plays a major role in HCV viral clearance.

Furthermore, in our study, we measured increased expression of IL-28 and IL-29 mRNA in the liver of patients infected with HCV in comparison to non-viral liver disease. IFN-λ mRNA was detectable in only 60.5% of the biopsies of non-viral liver disease but in 88.9% of the livers with HCV infection suggesting an essential role of HCV in the regulation of IFN-λ gene transcription. Further studies need to determine if the IFN-λ upregulation has a significant influence on the clinical presentation and the outcome of HCV infection. It may be hypothesized that patients with higher intrinsic IFN-λ expression show a better HCV clearance. This hypothesis is supported by the fact that lower IL-28A/B mRNA expression has been observed in non-responders to IFN-α therapy [51], [52]. Moreover, HCV patients with low endogenous IFN-λ expression might benefit more from a novel treatment with pegylated IL-29 than those with high IFN-λ levels.

The up-regulation of IFN-λ gene expression has also been described for other viral infections [1], [2], [8], [15], [53]. In contrast, the study of Mihm et al. shows that IFN-λ expression in the liver is similar in non-viral liver disease and HCV infection [54]. This difference to our study might be due to their small sample size in the non-viral liver disease group (8 samples) which included one outlier. However, there was a higher expression in HCV infection in comparison to healthy control liver tissue [54]. Moreover, they found higher IFN-λ mRNA expression in PBMC of HCV patients in comparison to healthy controls [54]. To our knowledge, IL-28A protein concentration has not been measured previously in the serum of HCV patients. Therefore, we developed an immunoluminometric assay which detected significantly higher IL-28A protein expression levels in the serum of HCV- or HBV-infected patients in comparison to healthy controls, but also in comparison to non-viral liver disease such as PBC. This suggests that IL-28A does not only have a “local” liver-specific role in the antiviral defense but also modulates the systemic antiviral immune response against HCV.

In further studies, it will be of great interest if the IL-28B-mediated gene expression and repression in hepatic cells resembles the pattern of IL-28A or IL-29 or is even different to both cytokines. Further investigations should elucidate if the different abilities of IL-28A and IL-29 to repress gene transcription have functional consequences in HCV infection *in vivo* as the replicon system represents only one single aspect of HCV life cycle. In addition, it will be of interest if these differences have practical impact in other viral infections *in vivo*, but also for the treatment of other diseases such as cancer. Given their ability to inhibit proliferation and to induce apoptosis [8], [55], IFN-λs have been also been discussed as a future cancer treatment option.

In summary, we have shown that both IL-28A and IL-29 induce expression of antiviral proteins, inhibit HCV replication and are up-regulated during viral infection with no major differences. However, in contrast to IL-29, IL-28A has the capacity to repress gene expression. Both cytokines are promising candidates for the treatment of HCV infection with likely low side effects on leukocytes. Nevertheless, further studies are needed to clarify which of the three IFN-λ cytokines is the most potent with the least amount of side effects.

## Supporting Information

Figure S1 **Functional categorization of IL-28A and IL-29 induced gene expression.** In all classifications, p-values <10^−5^ vs. the distribution of all genes on the microarray chip were considered as significant enrichment. Main classification groups are depicted in bold letters, while subgroups are written with normal letters. (A) Following IL-28A and IL-29 stimulation, genes of the biological processes of immunity and defense (with its subgroup interferon-mediated immunity) are significantly enriched. Proteolysis gene expression is significantly enriched only by IL-28A. In the legend, the classes are listed in a clock-wise order, starting at the “12 o'clock” position. (B) IL-28A and IL-29 both significantly enrich genes with the molecular functions of ligases, especially ubiquitin protein ligases. In the legend, the classes are listed in a clock-wise order, starting at the “12 o'clock” position.
(TIF)

Figure S2 **Functional categorization of IL-28A repressed gene expression.** (A) In IL-28A-treated samples, the down-regulated genes are enriched in the biological process of mRNA transcription regulation (for color chart legend, see Figure S1A). IL-29 did not down-regulate genes significantly (data not shown). (B) The molecular functions of IL-28A down-regulated genes comprise mainly of nucleic acid binding proteins and of homeobox transcription factors (for color chart legend, see Figure S1B).
(TIF)
