# Supplementary figures and images for: Comparative Analysis of the Lambda-Interferons IL-28A and IL-29 regarding Their Transcriptome and Their Antiviral Properties against Hepatitis C Virus
Source: PLoS One. 2010 Dec 8;5(12):e15200. doi: 10.1371/journal.pone.0015200 (PMC2999541; doi:10.1371/journal.pone.0015200)

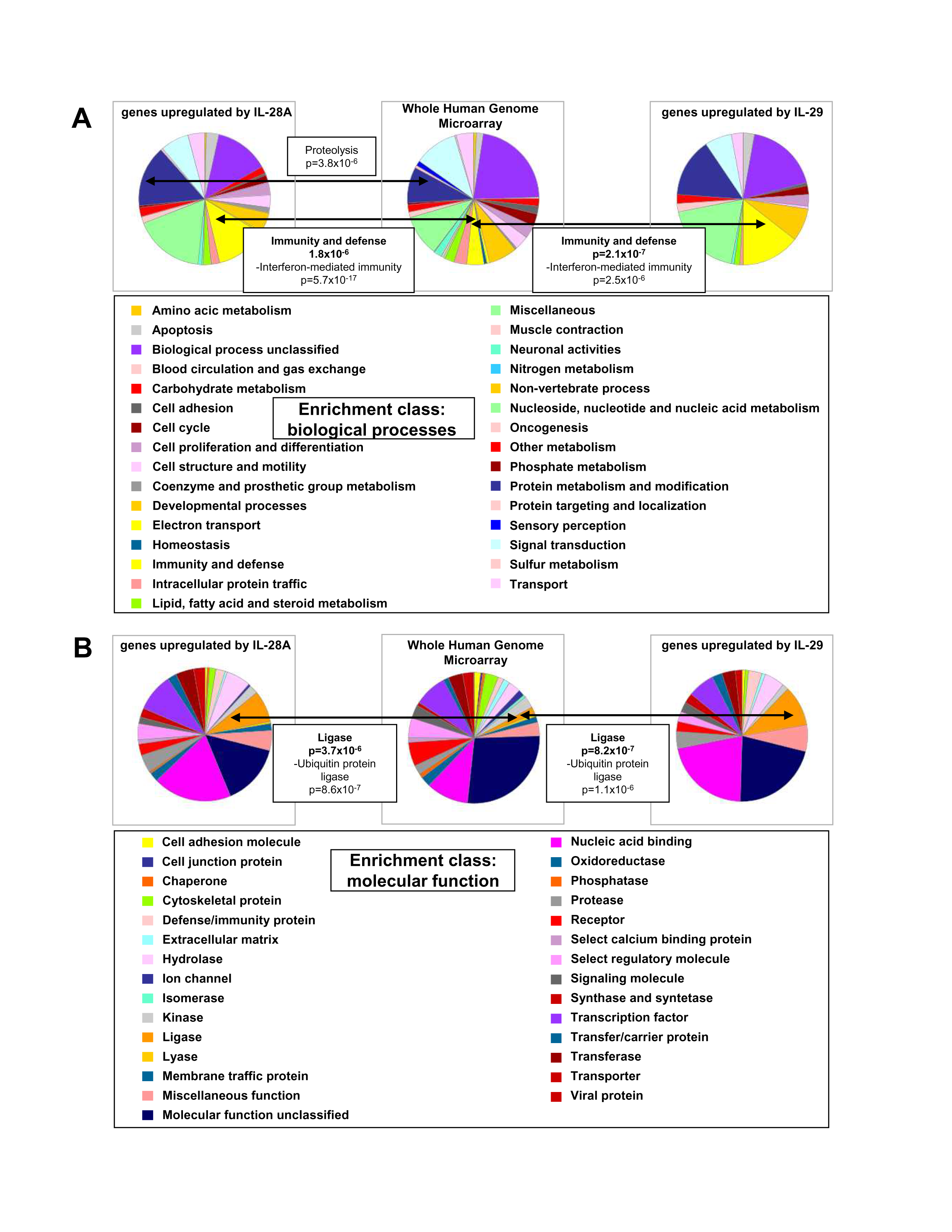

Supplement: Figure S1 — Functional categorization of IL-28A and IL-29 induced gene expression. In all classifications, p-values <10−5 vs. the distribution of all genes on the microarray chip were considered as significant enrichment. Main classification groups are depicted in bold letters, while subgroups are written with normal letters. (A) Following IL-28A and IL-29 stimulation, genes of the biological processes of immunity and defense (with its subgroup interferon-mediated immunity) are significantly enriched. Proteolysis gene expression is significantly enriched only by IL-28A. In the legend, the classes are listed in a clock-wise order, starting at the “12 o'clock” position. (B) IL-28A and IL-29 both significantly enrich genes with the molecular functions of ligases, especially ubiquitin protein ligases. In the legend, the classes are listed in a clock-wise order, starting at the “12 o'clock” position. (TIF) [file pone.0015200.s001.tif]

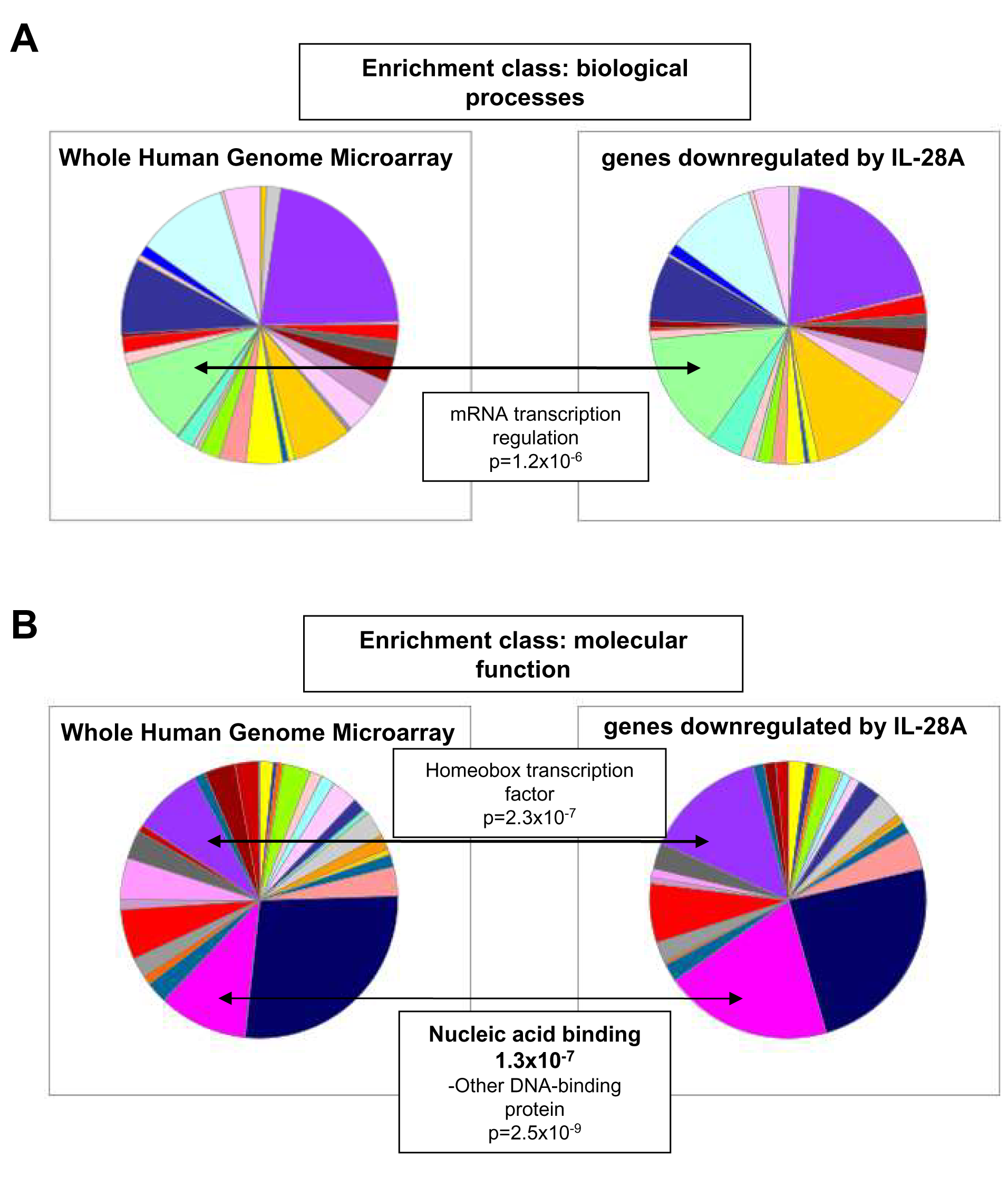

Supplement: Figure S2 — Functional categorization of IL-28A repressed gene expression. (A) In IL-28A-treated samples, the down-regulated genes are enriched in the biological process of mRNA transcription regulation (for color chart legend, see Figure S1A). IL-29 did not down-regulate genes significantly (data not shown). (B) The molecular functions of IL-28A down-regulated genes comprise mainly of nucleic acid binding proteins and of homeobox transcription factors (for color chart legend, see Figure S1B). (TIF) [file pone.0015200.s002.tif]
